# Randomized clinical trial of single skin sterilization with a povidone–iodine applicator *versus* conventional skin sterilization in abdominal surgery

**DOI:** 10.1002/bjs5.50144

**Published:** 2019-02-27

**Authors:** Y. Kambara, K. Hiramatsu, T. Kato, Y. Sibata, M. Yoshihara, T. Aoba, T. Aiba, N. Yamaguchi, T. Kamiya

**Affiliations:** ^1^ Department of General Surgery Toyohashi Municipal Hospital Toyohashi Japan; ^2^ Department of Gastrointestinal Surgery Japanese Red Cross Nagoya First Hospital Nagoya Japan; ^3^ Division of Surgical Oncology, Department of Surgery Nagoya University Graduate School of Medicine Nagoya Japan; ^4^ Department of Surgery Nagoya Ekisaikai Hospital Nagoya Japan

## Abstract

**Background:**

The efficacy of widely used povidone–iodine applicators for skin sterilization in abdominal surgery is unclear. The aim of this trial was to evaluate whether sterilization with a povidone–iodine applicator was not inferior to a conventional sterilization method.

**Methods:**

Patients undergoing elective abdominal surgery were assigned randomly to receive single sterilization with the applicator or conventional sterilization. The primary endpoint was wound infection rate. Secondary endpoints were rate of organ/space surgical‐site infection (SSI), adverse effects of povidone–iodine, amount of povidone–iodine used and total cost of sterilization.

**Results:**

Of 498 patients eligible for the study between April 2015 and September 2017, 240 were assigned and analysed in the applicator group and 246 in the conventional group. Wound infection was detected in 16 patients (6·7 per cent) in the applicator group and 16 (6·5 per cent) in the conventional group (absolute difference 0·0016 (90 per cent c.i. −0·037 to 0·040) per cent; *P* = 0·014 for non‐inferiority). There was no difference between the groups in the organ/space SSI rate (11 patients (4·6 per cent) in the applicator group and 16 (6·5 per cent) in the conventional group. Both the amount of povidone–iodine used and the total cost of sterilization were higher in the conventional group than in the applicator group (median 76·7 *versus* 25 ml respectively, *P* < 0·001; median €7·0 *versus* €6·4, *P* < 0·001). Skin irritation was detected in three patients in the conventional group.

**Conclusion:**

In abdominal surgery, this povidone–iodine applicator was not inferior to conventional sterilization in terms of the wound infection rate, and it is cheaper. Registration number: UMIN000018231 (http://www.umin.ac.jp/ctr/).

## Introduction

Skin sterilization before abdominal surgery is traditionally performed using a large amount of iodine antiseptic solution loaded on to surgical swabs or cotton balls. There is no clear evidence for the amount of disinfectant and number of swabs or balls used in existing guidelines^1^, ^2^. Large amounts of iodine disinfectant may cause adverse effects such as contact dermatitis and chemical burns^3^, ^4^, ^5^, ^6^, ^7^, ^8^, ^9^, ^10^, and may be unnecessarily wasteful. Povidone–iodine applicators have been approved by the Food and Drug Administration and are popular in the USA. They have several advantages, including sterile preparation, simplicity and low cost. However, the efficacy of these applicators in real clinical settings has not yet been demonstrated clearly. The aim of this study was to assess the non‐inferiority of a povidone–iodine applicator with a single sterilization using a small amount of solution *versus* conventional sterilization using a large amount of iodine disinfectant in patients undergoing elective abdominal surgery.

## Methods

The study was conducted with the approval of the ethics committee of Toyohashi Municipal Hospital, Toyohashi City, Aichi Prefecture, Japan. Written informed consent was obtained from all participants. The trial is registered with the UMIN Clinical Trials Registry (UMIN000018231).

The primary endpoint of this prospective RCT was the wound infection rate. Secondary endpoints were the rates of organ/space surgical‐site infection (SSI), the adverse effects of povidone–iodine, the amount of povidone–iodine used and the total cost of sterilization.

Patients who underwent elective abdominal surgery between April 2015 and September 2017 at Toyohashi Municipal Hospital, a district general hospital of Aichi prefecture in Japan, were enrolled in the study. Exclusion criteria were: disinfection site not being flat, such as abdominoperineal resection of the rectum and operations to treat oesophageal cancer; high risk of wound infection (any operation involving the creation of an artificial anus or its closure); or surgery being performed as part of another clinical trial (robot‐assisted operations, surgery inserting synthetic materials or operations where there was likely to be a different observation period for wound infection, such as inguinal hernia repair).

### Patient randomization

Eligible patients were randomly assigned 1 : 1 to either the applicator or the conventional sterilization group. Five hundred sealed opaque envelopes were prepared, numbered 1–250 twice, and distributed randomly.

After obtaining informed consent before surgery for participation in the study, an envelope was opened at the trial centre by one of the surgeons performing the operation. The patient's name, hospital identification number and allocated group were noted on the designation form, which was placed immediately in the designated box at the trial centre. Patients were blinded regarding their allocation. Neither the surgeons nor doctors assessing for wound infection were blinded to the patients' group allocation.

### Perioperative protocols and surgical procedures

Patients scheduled for lower digestive tract surgery underwent mechanical bowel preparation with magnesium citrate and sennosides at 15·00 and 21·00 hours respectively on the day before surgery. Other patients were prescribed only sennosides at 21·00 hours on the day before surgery. Patients took a bath or a shower the day before or on the morning of the operation. All hair within the proposed surgical area was clipped using electrical hair‐clippers after induction of anaesthesia, but before skin sterilization. Systematic scrubbing of the expected surgical area with antiseptic soap was not performed, in accordance with the local surgical guideline^11^. Perioperative prophylactic antibiotics were given 30 min before skin incision, and additional doses were administered every 3 h during surgery until skin closure. No additional doses of antibiotics were given after the operation was completed. Antibiotics were selected in accordance with their bacterial sensitivity: cefazolin was used for all upper gastrointestinal and hepatobiliary–pancreatic operations, and cefmetazole for lower intestinal surgery. Metronidazole was selected for patients with a documented cephalosporin allergy.

**Figure 1:**
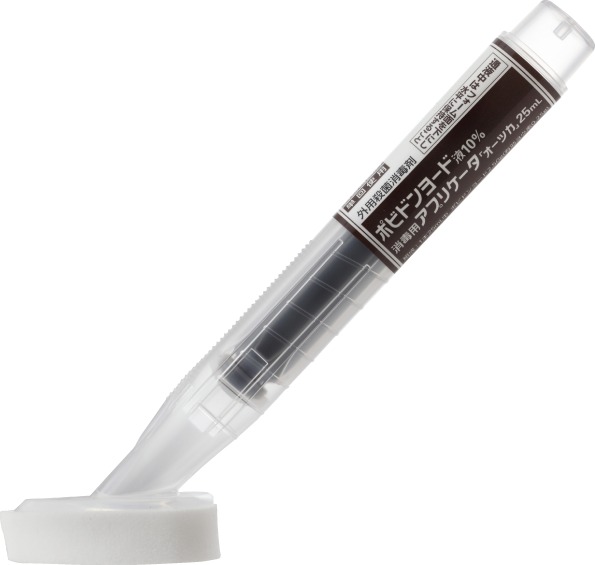
Povidone–iodine solution 10 per cent Antiseptic Applicator Otsuka 25 ml® (Otsuka Pharmaceutical, Tokyo, Japan)

Skin sterilization was commenced immediately after induction of anaesthesia. A surgeon who did not attend the operation sterilized the surgical field without scrubbing it with antiseptic soap, according to the local surgical guideline^11^. Patients in the applicator group received disinfection in a single outward spiralling movement from the centre of the abdomen using a povidone–iodine applicator (povidone–iodine solution 10 per cent Antiseptic Applicator Otsuka 25 ml®; Otsuka Pharmaceutical, Tokyo, Japan) (*Fig*. 1). Patients in the conventional group received disinfection via at least three outward spiralling movements from the centre of the abdomen, using povidone–iodine 10 per cent disinfectant and cotton balls. The amount of povidone–iodine disinfectant used in the conventional group was at least 75 ml, because this amount was judged to be the minimum required to wet sufficiently five cotton balls with a diameter of 3 cm in the cotton ball container at the authors' institution. If the operator determined that this amount was not enough, a further 25 ml disinfectant was added for each additional cotton ball required. The total amount of disinfectant used was recorded. Surgery was started when the povidone–iodine had dried, after draping the cleaned area appropriate for that operation. Surgical wounds were covered with disposable gauze for open abdominal surgery and with disposable wound protectors for laparoscopic abdominal surgery (SurgiSleeve™ Wound Protector; Medtronic, Dublin, Ireland). Where there was a perceived risk of anastomotic and/or bile leakage, closed intra‐abdominal drains were placed. Once all gloves had been changed, the fascia was closed with monofilament absorbable sutures. Incision sites were washed with saline, and subcutaneous and skin sutures were placed. Subcutaneous drains were not used. Incision sites were covered with sterile dressings, which were removed within 48 h. Intra‐abdominal drains were removed 1–5 days after operation. All procedures were performed or supervised by at least one of the eight general surgeons, each of whom had performed more than 500 surgical interventions and was board‐certified by the Japanese Surgical Society.

### Determination of wound and surgical‐site infections

Wound infection was defined as a superficial or deep incisional SSI according to guidelines issued by the US Centers for Disease Control and Prevention (CDC)^12^. Briefly, criteria for superficial and deep incisional SSIs were infections occurring at the incision site within 30 days of the procedure, involving the skin, subcutaneous tissue, muscle and fascia but not the organ/space, together with at least one of the following: purulent drainage from the incision; an organism isolated from culture of fluid from the incision; incisional pain, tenderness, localized swelling, redness or heat; and an incision that dehisced spontaneously or was opened deliberately by a surgeon in the presence of the signs and symptoms of infection described above. The criteria for organ/space SSIs were infection occurring within 30 days of the procedure in any part of the anatomy that was opened or manipulated during the operation other than the incisional site, together with at least one of the following: purulent fluid from a drain placed in the organ/space; an organism isolated from culture of fluid from the organ/space; abscess or other evidence of infection involving the organ/space found on direct examination, during reoperation, or by histopathological or radiological examination; and diagnosis of an organ/space SSI by a surgeon. Patients' wounds were observed by surgeons and nurses daily during hospitalization, and examined by surgeons 30 days after surgery.

### Sample size

The predetermined non‐inferiority margin was an absolute wound infection rate in the applicator group 5 per cent higher than that in the conventional group. Assuming a one‐sided α of 0·05, a power of 80 per cent and an expected 5 per cent incidence of wound infection in both groups, 235 patients per group were needed (Dunnett–Gent test^13^). Assuming an 8 per cent dropout rate, the planned required sample size was 253 patients per group. The non‐inferiority margin of 5 per cent was set for the primary endpoint only. Non‐inferiority was to be judged when the upper limit of the 90 per cent c.i. of the absolute difference in wound infection rate between the groups was lower than the predetermined non‐inferiority margin of 5 per cent.

### Statistical analysis

Continuous variables were analysed using Student's *t* test, and categorical variables with Pearson's χ^2^ or Fisher's exact test, as appropriate. The primary endpoint was analysed with the Dunnett–Gent test for evaluating non‐inferiority of the applicator group *versus* the conventional group. Statistical analysis was performed using R version 3.4.4 (R Foundation for Statistical Computing, Vienna, Austria) at a significance level of *P* < 0·050.

## Results

**Figure 2:**
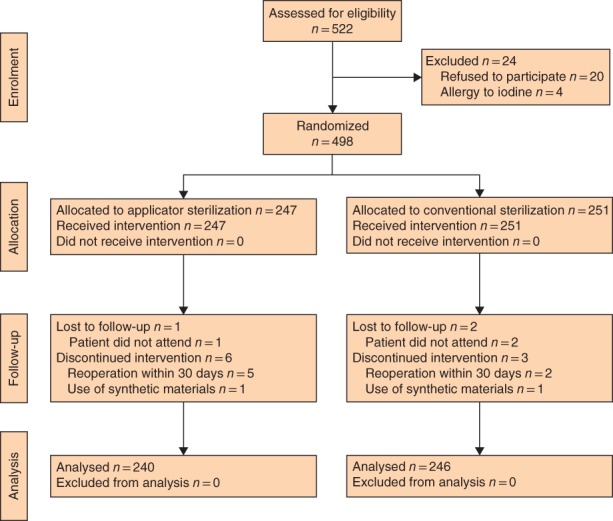
CONSORT diagram for the trial

From April 2015 to September 2017, 522 patients were initially considered eligible, but 20 patients did not agree to participate and four had a known iodine allergy. A total of 498 patients who underwent elective abdominal surgery agreed to participate and were eligible for enrolment. Of these, 247 and 251 patients were assigned to the applicator and conventional group respectively. After randomization, 12 patients were excluded; seven had reoperation within 30 days, two had synthetic materials inserted and three were lost to follow‐up, leaving 240 and 246 patients in the applicator and conventional group respectively, for final analysis (*Fig*. 2). All patients received the planned disinfection and were followed up for 30 days after surgery.

Patient and operation characteristics of the two groups were well balanced at baseline (*Tables* 1 and 2).

**Table 1:** Patient characteristics

	Applicator group (*n* = 240)	Conventional group (*n* = 246)
Age (years)*	66 (25–90)	68 (18–90)
Sex ratio (M : F)	131 : 109	147 : 99
BMI (kg/m^2^)*	22·8 (12·3–43·9)	22·9 (13·8–40·6)
Diabetes mellitus	32 (13·3)	28 (11·4)
Liver cirrhosis	11 (4·6)	8 (3·3)
Chronic obstructive pulmonary disease	0 (0·0)	2 (0·8)
Dialysis	2 (0·8)	1 (0·4)
Ischaemic heart disease	5 (2·1)	10 (4·1)
Steroid use	3 (1·3)	5 (2·0)
Immunosuppressant	1 (0·4)	4 (1·6)
Anticancer agent	1 (0·4)	2 (0·8)

Values in parentheses are percentages unless indicated otherwise; *values are median (range).

**Table 2:** Operations

	Applicator group (*n* = 240)	Conventional group (*n* = 246)
Upper digestive tract	72 (30·0)	62 (25·2)
Distal gastrectomy	41	36
Total gastrectomy	16	21
Partial gastrectomy	6	1
Remnant gastrectomy	2	3
Gastrojejunal bypass	7	1
Lower digestive tract	61 (25·4)	68 (27·6)
Ileocaecal resection	20	25
Right hemicolectomy	10	8
Transverse colectomy	5	7
Left hemicolectomy	6	2
Sigmoidectomy	10	13
Resection of rectum	6	6
Appendicectomy	2	4
Small intestinal resection	1	3
Ileocolic bypass	1	0
Hepatic, biliary, pancreatic, splenic surgery	111 (46·3)	107 (43·5)
Liver resection	26	15
Bile duct resection	3	5
Cholecystectomy	72	68
Pancreatic resection	8	17
Splenectomy	2	0
Other	0	2
Other operations	5 (2·1)	12 (4·9)
Abdominal lymph node biopsy	2	3
Intra‐abdominal tumour resection	2	9
Exploratory laparotomy	1	0
Laparoscopic surgery	120 (50·0)	119 (48·4)
Digestive tract reconstruction	127 (52·9)	135 (54·9)
Wound cleaning	132 (55·0)	136 (55·3)
Duration of surgery (min)*	158 (32–510)	176·5 (19–550)
Blood loss (ml)*	42·5 (0–1656)	44·0 (0–2528)

Some patients underwent multiple operations. Values in parentheses are percentages unless indicated otherwise; *values are median (range).

Wound infection was detected in 16 patients (6·7 per cent) in the applicator and 16 (6·5 per cent) in the conventional group (absolute difference 0·0016 (90 per cent c.i. −0·037 to 0·040) per cent)), confirming that the applicator method was not inferior to conventional sterilization (*P* = 0·014) (*Table* 3).

**Table 3:** Outcomes

	Applicator group (*n* = 240)	Conventional group (*n* = 246)	Absolute difference (%)†	Odds ratio‡	*P* §
Wound infection	16 (6·7)	16 (6·5)	0·0016 (−0·037, 0·040)		0·014¶
Organ/space infection	11 (4·6)	16 (6·5)		0·69 (0·31, 1·52)	0·421
Anastomotic leakage	1 (0·4)	2 (0·8)		0·51 (0·05, 5·67)	0·998
Intra‐abdominal abscess	2 (0·8)	1 (0·4)		2·06 (0·19, 22·86)	0·624
Pancreatic fistula	5 (2·1)	12 (4·9)		0·41 (0·14, 1·19)	0·128
Volume of disinfectant (ml)*	25	76·7 (75–100)		–	< 0·001#
Skin disorder by disinfection	0 (0)	3 (1·2)		–	0·129
Cost of sterilization (€)*	6·4	7·0 (6·9–7·2)		–	< 0·001#

Values in parentheses are percentages unless indicated otherwise; *values are median (range); †values in parentheses are 90 per cent c.i. for non‐inferiority; ‡values in parentheses are 95 per cent confidence intervals. §Pearson's χ^2^ or Fisher's exact test, except ¶Dunnett–Gent test (one‐sided for non‐inferiority) and #Student's *t* test.

Organ/space SSI was detected in 11 patients (4·6 per cent) in the applicator group and 16 (6·5 per cent) in the conventional group (*P* = 0·421). Median volumes of povidone–iodine used in the applicator and conventional group were 25 and 76·7 ml respectively (*P* < 0·001). The total cost of sterilization in the conventional group was calculated by the sum of the costs of disinfectant and disinfection kits; median total cost was €6·4 in the applicator group and €7·0 (range 6·9–7·2) in the conventional group (*P* < 0·001). Although not significantly different, the only adverse effect from povidone–iodine administration was skin irritation, seen only in the conventional group (3 patients, 1·2 per cent) (*Table* 3).

## Discussion

This study compared a povidone–iodine applicator method using a small amount of solution in abdominal surgery with a conventional sterilization method. Applicator sterilization was not inferior to conventional sterilization method in terms of the wound infection rate.

Various reports on wound infection control have been published, and guidelines have also been provided by the CDC^1^ and the UK National Institute for Health and Care Excellence^2^. Regarding skin sterilization before surgery, some studies^14^, ^15^, ^16^ have shown efficacy for various types of disinfectant, but there remains inadequate evidence with respect to disinfection procedures and amounts of disinfectant used.

Two differences were found between the povidone–iodine applicator with single sterilization and the conventional sterilization method: a lesser volume of disinfectant was used in the applicator group, and the equipment employed was different for the two methods. Although the amount of disinfectant was significantly lower in the applicator group, wound infection rates were no different: equivalent skin disinfection was achieved with 25 ml povidone–iodine using an applicator, without any preprocedural skin scrubbing with disinfectant soap. The value of presurgical skin scrubbing for the prevention of SSI is still controversial^17^. To simplify and minimize as many factors as possible to compare the power of sterilization, only povidone–iodine was examined in this study. The surgical site was sterilized with a single outward spiralling movement from the centre of the abdomen in the applicator group, suggesting it is unnecessary to sterilize the same site repeatedly, as was done in the conventional group. Some consideration should also be given to the use of large volumes of povidone–iodine as this can result in chemical burns, not simply allergic reactions^3^, ^4^, ^5^, ^6^, ^7^, ^8^, ^9^, ^10^. In the present study, three patients in the conventional group had a skin reaction.

The equipment differed between the applicator and conventional methods. The foam part of the applicator tip, which is in contact with the skin, is made of polyethylene, as according to the manufacturer it has a flat memorized shape. Compared with cotton balls used in the conventional method, it may be that the applicator tip can make more uniform contact with the skin surface, with disinfectant discharged at a uniform rate. For uneven sterilization surfaces such as the shoulder, a previous study^18^ reported a tendency for insufficient application when sterilizing with an applicator. However, this does not appear to be an issue when using the applicator on a relatively flat surface, such as the abdomen as in the present study, and a previous study^19^ used applicator preparations successfully for preoperative skin disinfection in clean‐contaminated surgery.

Several limitations of this study should be acknowledged. The study took place in a single institution, with potential bias in terms of evaluating efficacy and safety. It was not double‐blinded; although patients were blinded, the surgeons were not because of the nature of the study. Even after the povidone–iodine had dried, the difference in appearance between the two procedures was evident for surgeons, and this may have affected their attitude towards handling the wound. The minimum amount of povidone–iodine used in the conventional group was set at 75 ml. As the volume of disinfectant used at the authors' institution had never been measured before this study, it is possible that this amount differed from the normal amount used elsewhere.

## References

[1] Berríos‐Torres SI , Umscheid CA , Bratzler DW , Leas B , Stone EC , Kelz RR *et al.*; Healthcare Infection Control Practices Advisory Committee. Centers for Disease Control and Prevention guideline for the prevention of surgical site infection, 2017. JAMA Surg 2017; 152: 784–791. 28467526 10.1001/jamasurg.2017.0904

[2] National Institute for Health and Care Excellence (NICE) . *Surgical Site Infections: Prevention and Treatment. Clinical Guideline CG74*; 2008. https://www.nice.org.uk/guidance/cg74 [accessed 28 June 2018].

[3] Corazza M , Bulciolu G , Spisani L , Virgili A . Chemical burns following irritant contact with povidone–iodine. Contact Dermatitis 1997; 36: 115–116. 9062757 10.1111/j.1600-0536.1997.tb00433.x

[4] Nahlieli O , Baruchin AM , Levi D , Shapira Y , Yoffe B . Povidone–iodine related burns. Burns 2001; 27: 185–188. 11226660 10.1016/s0305-4179(00)00081-4

[5] Lowe DO , Knowles SR , Weber EA , Railton CJ , Shear NH . Povidone–iodine‐induced burn: case report and review of the literature. Pharmacotherapy 2006; 26: 1641–1645. 17064209 10.1592/phco.26.11.1641

[6] Kara A , Tezer H , Devrim I , Cengiz AB , Secmeer G . Chemical burn: a risk with outdated povidone iodine. Pediatr Dermatol 2007; 24: 449–450. 17845191 10.1111/j.1525-1470.2007.00492.x

[7] Murthy MB , Krishnamurthy B . Severe irritant contact dermatitis induced by povidone iodine solution. Indian J Pharmacol 2009; 41: 199–200. 20523874 10.4103/0253-7613.56069 PMC2875742

[8] Rees A , Sherrod Q , Young L . Chemical burn from povidone–iodine: case and review. J Drugs Dermatol 2011; 10: 414–417. 21455553

[9] Borrego L , Hernández N , Hernández Z , Peñate Y . Povidone–iodine induced post‐surgical irritant contact dermatitis localized outside of the surgical incision area. Report of 27 cases and a literature review. Int J Dermatol 2016; 55: 540–545. 26475438 10.1111/ijd.12957

[10] Okano M . Irritant contact dermatitis caused by povidone–iodine. J Am Acad Dermatol 1989; 20: 860. 10.1016/s0190-9622(89)80129-x 2715439

[11] Harihara Y. Syujyutunojissenngaidorainn‐Syujyututokannsennbousi‐Syujytubuikannsennbousi. J Jpn Assoc Oper Room Technol 2013; 34: 5058–5070 (in Japanese).

[12] Mangram AJ , Horan TC , Pearson ML , Silver LC , Jarvis WR . Guideline for prevention of surgical site infection, 1999. Hospital Infection Control Practices Advisory Committee. Infect Control Hosp Epidemiol 1999; 20: 250–278. 10219875 10.1086/501620

[13] Dunnett CW , Gent M . An alternative to the use of two‐sided tests in clinical trials. Stat Med 1996; 15: 1729–1738. 8870155 10.1002/(SICI)1097-0258(19960830)15:16<1729::AID-SIM334>3.0.CO;2-M

[14] Noorani A , Rabey N , Walsh SR , Davies RJ . Systematic review and meta‐analysis of preoperative antisepsis with chlorhexidine *versus* povidone–iodine in clean‐contaminated surgery. Br J Surg 2010; 97: 1614–1620. 20878942 10.1002/bjs.7214

[15] Lee I , Agarwal RK , Lee BY , Fishman NO , Umscheid CA . Systematic review and cost analysis comparing use of chlorhexidine with use of iodine for preoperative skin antisepsis to prevent surgical site infection. Infect Control Hosp Epidemiol 2010; 31: 1219–1229. 20969449 10.1086/657134 PMC3833867

[16] Davies BM , Patel HC . Systematic review and meta‐analysis of preoperative antisepsis with combination chlorhexidine and povidone–iodine. Surg J (N Y) 2016; 2: e70–e77. 28824994 10.1055/s-0036-1587691 PMC5553484

[17] Lefebvre A , Saliou P , Mimoz O , Lucet JC , Le Guyader A , Bruyère F *et al.*; French Study Group for the Pre‐operative Prevention of Surgical Site Infections . Is surgical site scrubbing before painting of value? Review and meta‐analysis of clinical studies. J Hosp Infect 2015; 89: 28–37. 25477062 10.1016/j.jhin.2014.10.004

[18] Syed UAM , Seidl AJ , Hoffman RA , Bianchini J , Beredjiklian PK , Abboud JA . Preoperative sterilization preparation of the shoulder: a comparative study evaluating gauze sponge and commercially available applicator prep stick. Arch Bone Jt Surg 2018; 6: 34–38. 29430493 PMC5799598

[19] Magalini S , Pepe G , Panunzi S , De Gaetano A , Abatini C , Di Giorgio A *et al.* Observational study on preoperative surgical field disinfection: povidone–iodine and chlorhexidine–alcohol. Eur Rev Med Pharmacol Sci 2013; 17: 3367–3375. 24379069

